# Possible Mechanism of Action of the Antiallergic Effect of an Aqueous Extract of *Heliotropium indicum* L. in Ovalbumin-Induced Allergic Conjunctivitis

**DOI:** 10.1155/2015/245370

**Published:** 2015-11-10

**Authors:** Samuel Kyei, George Asumeng Koffuor, Paul Ramkissoon, Samuel Abokyi, Osei Owusu-Afriyie, Eric Addo Wiredu

**Affiliations:** ^1^Discipline of Optometry, School of Health Sciences, College of Health, University of KwaZulu-Natal, Durban 4041, South Africa; ^2^Department of Optometry, School of Allied Health Sciences, University of Cape Coast, Cape Coast, Ghana; ^3^Department of Pharmacology, Faculty of Pharmacy and Pharmaceutical Sciences, Kwame Nkrumah University of Science and Technology, Kumasi, Ghana; ^4^Department of Pathology, Komfo Anokye Teaching Hospital, Kumasi, Ghana

## Abstract

*Heliotropium indicum* is used traditionally as a remedy for conjunctivitis in Ghana. This study therefore evaluated the antiallergic potential of an aqueous whole plant extract of *Heliotropium indicum* (HIE) in ovalbumin-induced allergic conjunctivitis and attempted to predict its mode of action. Clinical scores for allergic conjunctivitis induced by intraperitoneal ovalbumin sensitization (100 : 10 *μ*g OVA/Al(OH)_3_ in phosphate-buffered saline [PBS]) and topical conjunctival challenge (1.5 mg OVA in 10 *μ*L PBS) in Dunkin-Hartley guinea pigs were estimated after a week's daily treatment with 30–300 mg kg^−1^ HIE, 30 mg kg^−1^ prednisolone, 10 mg kg^−1^ chlorpheniramine, or 10 mL kg^−1^ PBS. Ovalbumin-specific IgG and IgE and total IgE in serum were estimated using Enzyme-Linked Immunosorbent Assay. Histopathological assessment of the exenterated conjunctivae was also performed. The 30 and 300 mg kg^−1^ HIE treatment resulted in a significantly (*p* ≤ 0.001) low clinical score of allergic conjunctivitis. Ovalbumin-specific IgG and IgE as well as total serum IgE also decreased significantly (*p* ≤ 0.01–0.001). The conjunctival tissue in HIE treated guinea pigs had mild mononuclear infiltration compared to the PBS-treated ones, which had intense conjunctival tissue inflammatory infiltration. HIE exhibited antiallergic effect possibly by immunomodulation or immunosuppression.

## 1. Introduction 

Allergic conjunctivitis is a common problem that ophthalmic practitioners have to deal with, almost on daily basis, as it affects nearly 40% of the populace in advanced nation [1, 2]. Various studies in Africa have indicated the prevalence of allergic conjunctivitis to range between 7.3 and 32% [3, 4].

The conjunctiva is a dynamic immunologic tissue that suffers lymphoid hyperplasia in reaction to a stimulant such as pollens, animal dander, and other environmental antigens [5]. Allergic conjunctivitis (AC), therefore, is clinically characterized by pruritus, hyperemia, chemosis, tearing, and photophobia [6]. These clinical symptoms are the reason for the high morbidity associated with AC and consequential impact on quality of life of AC victims [7, 8]. Due to the large burden of AC and its spate of increase all over the world and across all ages, it possesses a great challenge to the health care resources of many countries [9, 10].

Allergic conjunctivitis is prompted by IgE-mediated immediate hypersensitivity reaction. Mast cell plays an important role in these allergic inflammations [11, 12]. Available medical therapies which include antihistamines, mast cell stabilizers, corticosteroids, nonsteroidal anti-inflammatory drugs, immunomodulatory agents, and allergen-specific immunotherapy could be rendered ineffective due to discomfort associated with administration of medications, intricacy of administration guidelines, perceived lack of efficacy by users, and/or adverse effects [13].

In the light of these bottlenecks associated with effective management of AC, current studies have focused attention on plants and natural products based therapeutic strategies in the bid to broaden treatment horizon, improve efficacy, and address safety concerns [14].* Heliotropium indicum* L. (Boraginaceae), also known as Indian heliotrope, is one such plant used in the traditional management of conjunctivitis [15, 16]. Although* H. indicum* is well-studied medicinal plant, its traditional use in treating ocular allergic inflammation is yet to be evaluated. This study therefore sought to evaluate the antiallergic effect and possible mechanism of action of whole plant aqueous extract of* Heliotropium indicum* L. in ovalbumin-induced allergic conjunctivitis in Dunkin-Hartley guinea pigs.

## 2. Materials and Methods

### 2.1. Plant Collection

*Heliotropium indicum* was collected from the botanical gardens of the University of Cape Coast, Cape Coast, in the Central Region of Ghana (5.1036°N, 1.2825°W) in November 2012. It was identified and authenticated by a botanist at the School of Biological Sciences, College of Agricultural and Natural Science, University of Cape Coast, Cape Coast, Ghana, where a voucher specimen with number 4873 has been deposited at the herbarium for future reference.

### 2.2. Preparation of the Aqueous Extract of* H. indicum* (HIE)

Whole plants of* H. indicum* were washed thoroughly with tap water and shade-dried. The dry plants were milled into coarse powder by a hammer mill (Schutte Buffalo, New York, NY). One and half kilograms of the plant powder was mixed with one liter of water. The mixture was Soxhlet-extracted at 80°C for 24 h. The aqueous extract obtained was freeze-dried (Hull Freeze-Dryer/Lyophilizer 140 SQ, Warminster, PA). The powder (yield 12.2%), labeled as HIE, was stored at 4°C and reconstituted in normal saline to the desired concentration for dosing in this study.

### 2.3. Drugs and Chemicals

Ovalbumin (OVA) (Cayla-InvivoGen, Toulouse, France), aluminum hydroxide (Merck, Darmstadt, Germany), chloroform (Sigma-Aldrich, USA), cetirizine (McNeil Consumer Healthcare, Washington, USA), and prednisolone (Taizhou Baida Pharmaceutical Chemical Co., Ltd., China) were some chemicals used in this study.

### 2.4. Animal and Husbandry

Dunkin-Hartley guinea pigs (weight 300 ± 25 g) were kept in the Animal House of the School of Biological Sciences, University of Cape Coast, Ghana. The experimental animals were housed in aluminum cages (34 cm × 47 cm × 18 cm) with soft wood shavings as bedding, under ambient laboratory conditions (temperature 28°C ± 2°C, relative humidity 60–70%, and a normal light-dark cycle). They were fed on a normal commercial pellet diet (Agricare Ltd., Kumasi, Ghana) and had access to water* ad libitum*.

### 2.5. Ethical and Biosafety Considerations

The study protocols were approved by the Institutional Review Board on Animal Experimentation of the Faculty of Pharmacy and Pharmaceutical Sciences, Kwame Nkrumah University of Science and Technology, Kumasi, Ghana (Ethical Clearance number FPPS/PCOL/0030/2013). All activities performed during the studies conformed to accepted principles for laboratory animal use and care (EU directive of 1986: 86/609/EEC) and Association for Research in Vision and Ophthalmology Statement for Use of Animals in Ophthalmic and Vision Research. Biosafety guidelines for protection of personnel in the laboratory were observed.

### 2.6. Preliminary Phytochemical Screening

Screening was performed on HIE to ascertain the presence of phytochemicals using standard procedures described by Harborne [17] and Kujur et al. [18].

### 2.7. Ovalbumin-Induction Allergic Conjunctivitis (OIAC)

OIAC was carried out as described by Shoji et al. [19] and Abokyi et al. [20]. Guinea pigs were sensitized by two intraperitoneal injections of 0.2 mL solution containing 100 *μ*g OVA and 0.01 mg aluminum hydroxide in phosphate buffer saline (pH 7.4) at an interval of 2 weeks. On day 8 after the sensitization, conjunctivitis was induced by topical instillation (challenge stage) of OVA (1.5 mg OVA in 10 *μ*L PBS) into the conjunctival sac of each eye. The topical challenge was repeated after 2 days. Physical and slit lamp (Marco II-B, Lombart Instrument, Japan) biomicroscopic ocular examinations were conducted. Hyperemia of palpebral conjunctiva, chemosis of bulbar conjunctiva, and lid swelling (clinical symptoms of AC) observed in the animal were an indication that AC had been induced.

### 2.8. Effect of HIE on OIAC

#### 2.8.1. Grouping and Dosing

The animals with conjunctivitis were put into six groups (*n* = 5). Groups I–III were treated with 30, 100, and 300 mg kg^−1^ HIE, respectively, Group IV was treated with 10 mg kg^−1^ chlorpheniramine, and Group V received 30 mg kg^−1^ prednisolone, while Group VI (control group) was treated with 10 mL kg^−1^ PBS. A normal control group, Group VII (no sensitization and challenge, no interventionary treatment), was kept under experimental condition. All treatments were* per os *and were started 24 h after the last topical challenge. Treatment was twice daily (12-hour interval) for one week.

#### 2.8.2. Clinical Assessment of HIE in OIAC

Clinical examination was performed on days 1, 3, 5, and 7 in the various groups of animals. Hyperemia of palpebral conjunctiva, chemosis of bulbar conjunctiva, and lid swelling were scored on a scale of 0–3. The total of clinical scores for hyperemia of palpebral conjunctiva, chemosis of bulbar conjunctiva, and lid swelling was also evaluated as an overall clinical score [21].

#### 2.8.3. Determination of Ovalbumin Specific IgE, IgG, and Total Serum IgE

Twenty-four hours after the last treatment had been administered, guinea pigs were anesthetized with chloroform and whole blood was collected from the jugular vein into pyrogenic free test tube (Indigo Instrument, Waterloo, Canada). Serum was prepared by centrifuging the clotted blood (temperature 25°C, speed 3000 g) for 5 min using a Mikro 220R machine (Hettich Zentrifuge, Tuttlingen, Germany). The serum was then subjected to Enzyme-Linked Immunosorbent Assay (ELISA) (MyBioSource, San Diego, CA, USA) as per the manufacturer's instructions briefly described. This employed the double-sandwich ELISA technique. The precoated antibodies were either one of the following: guinea pig OVA sIgG, sIgE, and IgE monoclonal antibody and the detecting antibodies, a biotin labeled polyclonal antibody. In each case test samples and biotin labeled antibody were added to ELISA plate microwells. These were washed out with phosphate buffered saline (PBS). Avidin-peroxidase conjugates were then added to the ELISA wells; tetramethylbenzidine (TMB) substrate was used for coloring and washed out. TMB turned blue after intermittent incubation and finally yellow under the action of acid. The absorbances were then read at 450 nm using an URIT-660 microplate reader (URIT Medical Electronic Co., Ltd., Guangxi, China). Each determination was in triplicate.

#### 2.8.4. Histopathological Assessment

The eyeballs together with the conjunctiva and lids of animals from the various groups were exenterated and fixed in 10% buffered formalin. Conjunctival tissue was stained with hematoxylin and eosin. Histopathological assessment was done by a specialist pathologist at Komfo Anokye Teaching Hospital, Kumasi, Ghana.

### 2.9. Statistical Analysis

The statistical analysis of data obtained was made using GraphPad Prism Version 5.0 (GraphPad Software, Inc., USA). Differences between treatment groups and the controls were estimated using One-Way Analysis of Variance (ANOVA) followed by Dunnett's Multiple Comparisons Test (post hoc test) at a confidence level of 95%. Probability values less than or equal to 5% (*p* ≤ 0.05) were considered significant.

## 3. Results

### 3.1. Phytochemical Screening

Preliminary phytochemistry showed that flavonoids, saponins, cyanogenic glycosides, sterols, tannins, and alkaloids were present in HIE (Table 1).

### 3.2. Effect of HIE on Clinical Signs of OIAC

**Figure 1:**
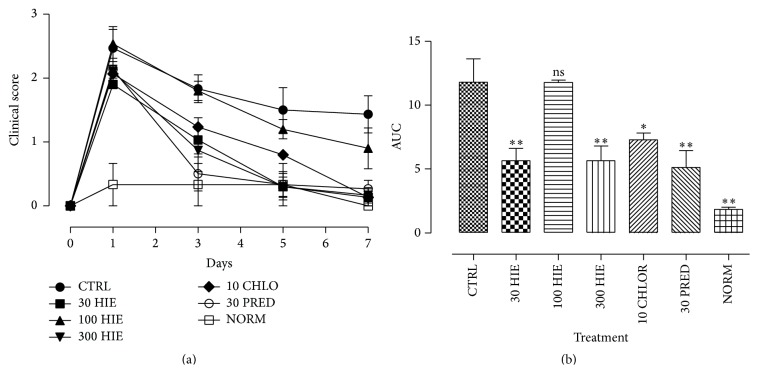
Time-course curves (a) and areas under the curve (b) for the effects of 30, 100, and 300 mg kg^−1^ of HIE and 10 mg kg^−1^ chlorpheniramine and 10 mg kg^−1^ prednisolone on OIAC in Dunkin-Hartley guinea pigs. Values plotted are mean ± SEM (*n* = 5). ^*∗*^ *p* ≤ 0.01; ^*∗∗*^ *p* ≤ 0.001, ANOVA followed by Dunnett's multiple comparisons test.

The clinical scores for OIAC indicated a U-shaped effect of HIE in mitigating the clinical signs of allergic conjunctivitis. The 30 and 300 mg kg^−1^ significantly (*p* ≤ 0.001) attenuated the clinical signs of allergic inflammation and not the 100 mg kg^−1^ (Figures 1(a) and 1(b)). Chlorpheniramine and prednisolone (reference drugs) significantly (*p* ≤ 0.01–0.001) attenuated the clinical signs of AC (Figures 1(a) and 1(b)).

### 3.3. HIE Effect on Sera OVA-Specific IgE, IgG, and Total IgE

**Figure 2:**
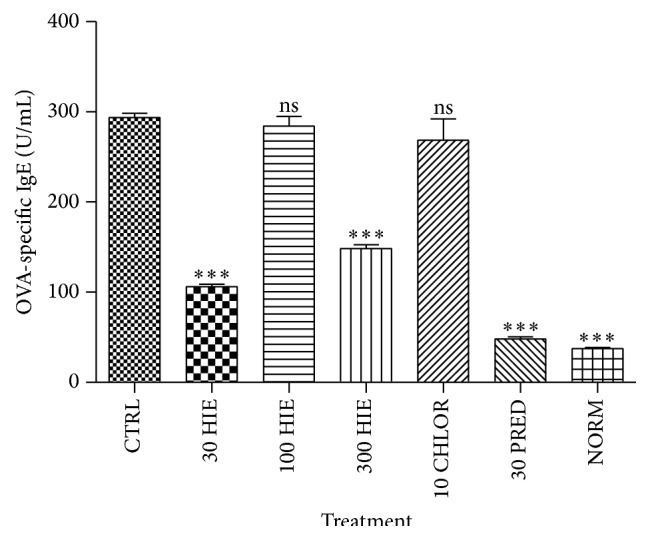
The effect of 30, 100, and 300 mg kg^−1^ HIE, 30 mg kg^−1^ prednisolone, and 10 mg kg^−1^ chlorpheniramine on OVA-specific serum IgE in OIAC in guinea pigs. ^*∗∗∗*^ *p* ≤ 0.001, ANOVA followed by Dunnett's post hoc test.

**Figure 3 fig3:**
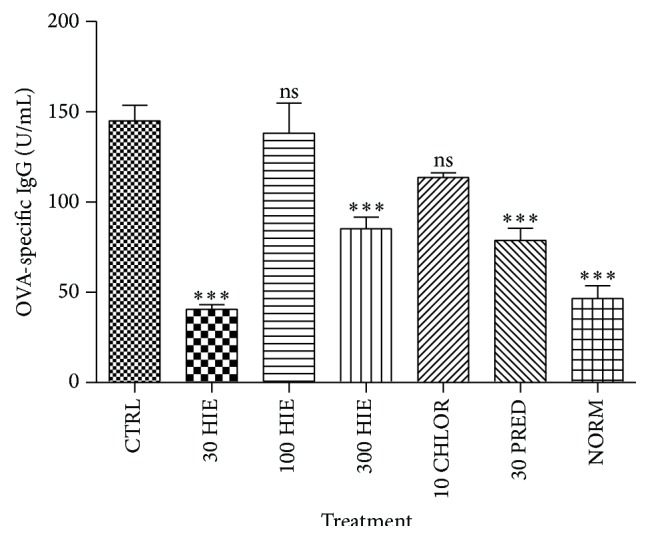
The effect of 30, 100, and 300 mg kg^−1^ HIE, 30 mg kg^−1^ prednisolone, and 10 mg kg^−1^ chlorpheniramine on OVA-specific serum IgG in OIAC in guinea pigs. ^*∗∗∗*^ *p* ≤ 0.001, ANOVA followed by Dunnett's post hoc test.

**Figure 4:**
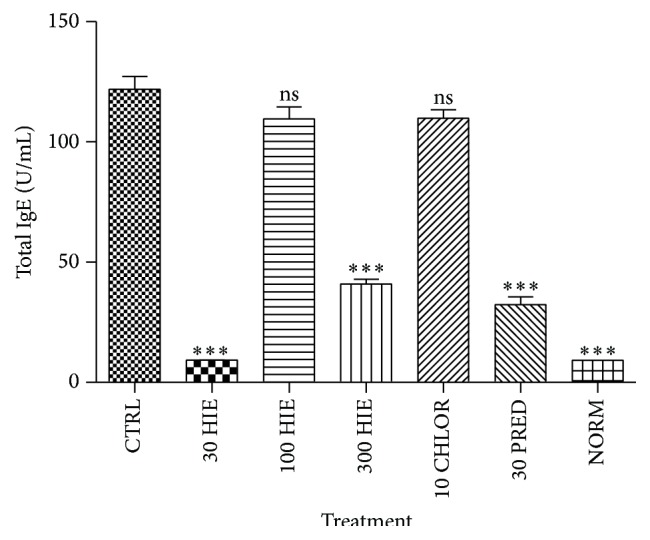
The effect of 30, 100, and 300 mg kg^−1^ HIE, 30 mg kg^−1^ prednisolone, and 10 mg kg^−1^ chlorpheniramine on total serum IgE in OIAC in guinea pigs. ^*∗∗∗*^ *p* ≤ 0.001, ANOVA followed by Dunnett's post hoc test.

HIE showed a U-shaped effect in decreasing (*p* ≤ 0.01–0.001) sera OVA-specific IgE, IgG, and total IgE antibodies in serum. Prednisolone caused significant reduction (*p* ≤ 0.001) in the immunoglobulin, but chlorpheniramine did not (Figures 2 [Fig fig3]–4).

### 3.4. Histopathological Assessment

**Figure 5:**
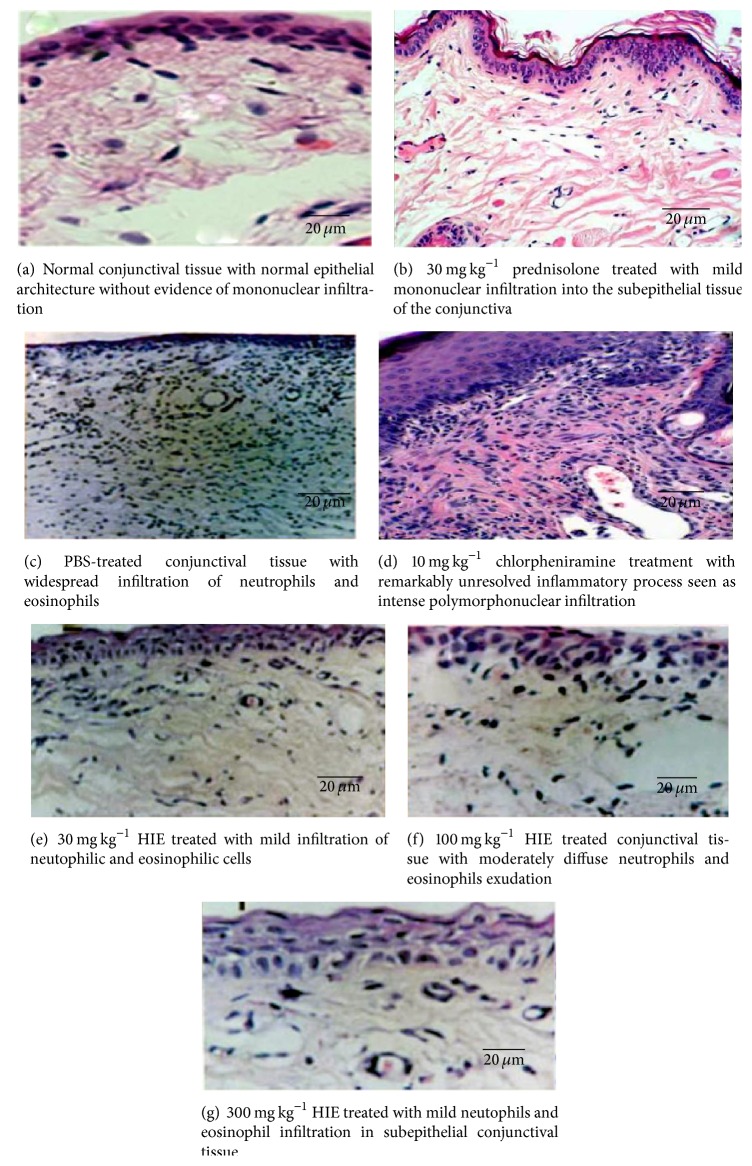
Photomicrographs of the conjunctival tissues in OIAC in guinea pigs treated with 30, 100, and 300 mg kg^−1^ HIE, 30 mg kg^−1^ prednisolone, 10 mg kg^−1^ chlorpheniramine, and PBS, compared to that of normal guinea pigs.

The histopathological assessment showed remarkable signs of mononuclear infiltration in conjunctival tissue of the control (PBS treated) group. Treatment with 30 and 300 mg kg^−1^ showed a reduction in mononuclear infiltrations but not the 100 mg kg^−1^ HIE treated group. Prednisolone reduced the infiltration much more than chlorpheniramine (Figure 5).

## 4. Discussion 

Ovalbumin-induced allergic conjunctivitis model in the guinea pig has been used in preclinical studies in screening for potential antiallergic agents [22]. It has been noted as an ideal model for both IgE mediated and non-IgE mediated allergic conjunctivitis [23]. This model of ocular allergic disease is typically related to type 1 hypersensitivity reactions. It is biphasic with the early-phase reaction driven primarily by mast cell degranulation and ensues right after exposure to the allergen; the late-phase reaction is marked by cell infiltration, mainly eosinophils, neutrophils, and lymphocytes, 6 to 24 hours after antigen application, corresponding to the clinical findings of allergic conjunctivitis [24]. Activation and conscription of inflammatory cells and the liberation of cytokines, chemokines, adhesion molecules, and proteases promote more serious chronic forms [25].

The low clinical scores obtained for the extract treated group justify the extract's potency in relieving the noisome symptoms associated with the underlying pathology of allergic conjunctivitis, the hallmark of most antiallergic agents. Although the U-shaped dose-response effect observed in clinical scores of ocular allergy remains unclear, it has been reported in several pharmacological investigations. This observation could be due to the inhibitory tendencies of the active phytochemical at that dose [26].

HIE reduced both allergen- (OVA-) specific IgG and IgE indicating a mechanistic deviation of activity from antihistaminic and mast cell stabilizing agents [20, 27]. Treatment with chlorpheniramine irrespective of the favorable clinical outcome regarding the resolution of allergic inflammation had no significant effect on allergen-specific IgG, IgE, and total sera IgE. This is because the release of histamine whose receptors are the target for antihistaminic agents lies downstream immunoglobulin production. This therefore accounts for its ability to attenuate the clinical signs of allergic inflammation [28]. The extract treatment causing relevant reduction in allergen-specific immunoglobulins was similar to the effect of the steroid treatment (prednisolone). This presupposes that the extract may have immunomodulatory and or immunosuppressive effect [13, 29].

A number of immunomodulatory compounds have been isolated from natural products [30, 31]. Antioxidant-rich extracts have been found relevant as immunomodulatory or immunosuppressive agents and have been mainly used in control of the immune response in conditions like transplantation, autoimmune disorders, and alleviation of allergic diseases [31]. Some studies have already reported the antioxidant properties of both extract and different fractions of* H. indicum* which in effect suggest that HIE's antiallergic inflammatory effect could probably be due to its rich antioxidant constituents such as flavonoids, alkaloids, and tannins [32–34].

HIE treatment reduced serum levels of IgE which is one of the necessary ingredients in the promulgation of allergic inflammatory processes [35]. When an allergen (e.g., ovalbumin) is taken up by antigen presenting cells (e.g., allergen-specific B cell), via the cell surface immunoglobulin receptor, processed fragments are then presented in the context of major histocompatibility class II (MHC class II) to Th2 cells recognizing the allergen-MHC II complex. Activation of the allergen-specific Th2 cells results in the expression of IL-4, IL-13, and CD154 and introduction of class switching to IgE. Class switching, a process involved in the biosynthesis of immunoglobulins, is driven by allergens [36]. Class switching is ushered by T cells signalling. Nevertheless, basophils express high levels of IL-4, IL-13, and CD154 after stimulation and it has been postulated to play a role in polyclonal amplification of IgE production and in the differentiation of Th2 cells [37]. These immunoglobulins bind to high-affinity IgE receptor; Fc*ε*R1 expressed on mast cells and basophils as tetramers (*αβγ*_2_) and on antigen presenting cells, at much lower levels, as trimers (*αγ*_2_) leading to degranulation of mast cells [38]. Studies have shown that the density of human basophil and mast cell Fc*ε*R1 expression is associated with serum IgE levels [39]. Mast cell degranulation is dependent on Syk kinase responsible for signaling proceedings subsequent to mast cells and basophils stimulation. This assertion is supported by recent finding indicating that Syk kinase deficient mast cells and basophils do not undergo degranulation after Fc*ε*R1 aggregation [40]. This therefore opens a window of opportunity for therapeutic exploration. The extract in this case indicates a mechanistic efficacy in reducing serum OVA allergen-specific and total IgE levels.

## 5. Conclusion

The aqueous whole plant extract of* Heliotropium indicum* exhibits antiallergic effect in ovalbumin-induced conjunctivitis in guinea pigs via a probable immunomodulating or immunosuppressive action supporting its traditional use in treatment of conjunctivitis.

## Figures and Tables

**Table 1:** Results obtained after preliminary phytochemical screening of HIE.

Phytochemical tested for	Results obtained
Anthraquinones	−
Tannins	+
Flavonoids	+
Alkaloids	+
Sterols	+
Glycosides	+
Saponins	+
Triterpenoids	−

“+” indicates presence; “**−**” indicates absence.
